# {μ-6,6′-Dimeth­oxy-2,2′-[propane-1,3-diylbis(nitrilo­methyl­idyne)]diphenolato}dimethano­ltrinitratonickel(II)europium(III) methanol disolvate

**DOI:** 10.1107/S1600536808041445

**Published:** 2008-12-20

**Authors:** Fei Liu

**Affiliations:** a The College of Chemical Engineering & Materials, Eastern Liaoning University, Liaoning 118003, People’s Republic of China

## Abstract

The title dinuclear complex, [EuNi(C_19_H_20_N_2_O_4_)(NO_3_)_3_(CH_3_OH)_2_]·2CH_3_OH, is isostructural with its Ni^II^/Pr^III^ analogue. The Ni^II^ ion is coordinated by two O atoms and two N atoms of a Schiff base ligand and by two methanol mol­ecules, forming a slightly distorted octa­hedral geometry. The Eu^III^ ion is coordinated by six O atoms from three chelating nitrate ligands and four O atoms from a Schiff base ligand, forming a distorted bicapped square-anti­prismatic environment. Inter­molecular O—H⋯O hydrogen bonds connect complexes and methanol solvent mol­ecules.

## Related literature

For the isostructural Ni^II^/Pr^III^ compound, see: Liu & Zhang (2008 ▶). For a related Cu^II^/Sm^III^ compound, see: Wang *et al.* (2008 ▶).
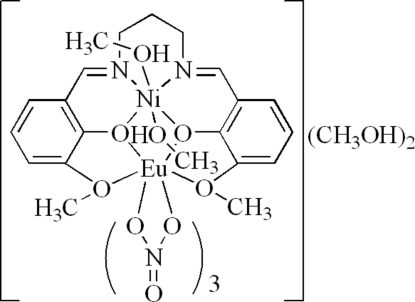


## Experimental

### 

#### Crystal data

[EuNi(C_19_H_20_N_2_O_4_)(NO_3_)_3_(CH_4_O)_2_]·2CH_4_OH
*M* *_r_* = 865.24
Monoclinic, 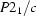

*a* = 13.062 (3) Å
*b* = 11.105 (2) Å
*c* = 22.122 (4) Å
β = 90.81 (3)°
*V* = 3208.3 (11) Å^3^
*Z* = 4
Mo *K*α radiation
μ = 2.61 mm^−1^
*T* = 291 (2) K
0.24 × 0.23 × 0.21 mm

#### Data collection

Rigaku R-AXIS RAPID diffractometer
Absorption correction: multi-scan (*ABSCOR*; Higashi, 1995 ▶) *T*_min_ = 0.572, *T*_max_ = 0.605
28507 measured reflections
7257 independent reflections
6231 reflections with *I* > 2σ(*I*)
*R*_int_ = 0.031

#### Refinement

*R*[*F* ^2^ > 2σ(*F* ^2^)] = 0.028
*wR*(*F* ^2^) = 0.060
*S* = 1.08
7257 reflections
430 parameters
6 restraints
H-atom parameters constrained
Δρ_max_ = 0.65 e Å^−3^
Δρ_min_ = −0.46 e Å^−3^

### 

Data collection: *RAPID-AUTO* (Rigaku, 1998 ▶); cell refinement: *RAPID-AUTO*; data reduction: *CrystalStructure* (Rigaku/MSC, 2002 ▶); program(s) used to solve structure: *SHELXS97* (Sheldrick, 2008 ▶); program(s) used to refine structure: *SHELXL97* (Sheldrick, 2008 ▶); molecular graphics: *SHELXTL* (Sheldrick, 2008 ▶); software used to prepare material for publication: *SHELXL97*.

## Supplementary Material

Crystal structure: contains datablocks global, I. DOI: 10.1107/S1600536808041445/bi2329sup1.cif

Structure factors: contains datablocks I. DOI: 10.1107/S1600536808041445/bi2329Isup2.hkl

Additional supplementary materials:  crystallographic information; 3D view; checkCIF report

## Figures and Tables

**Table 1:** Hydrogen-bond geometry (Å, °)

*D*—H⋯*A*	*D*—H	H⋯*A*	*D*⋯*A*	*D*—H⋯*A*
O14—H24⋯O16	0.83	1.86	2.657 (4)	162
O15—H25⋯O6^i^	0.82	2.28	3.092 (4)	174
O16—H16⋯O17^ii^	0.82	1.94	2.715 (6)	157
O17—H17⋯O13	0.82	2.15	2.923 (5)	158

Symmetry codes: (i) 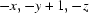; (ii) 
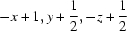
.

**Fig. 1.**
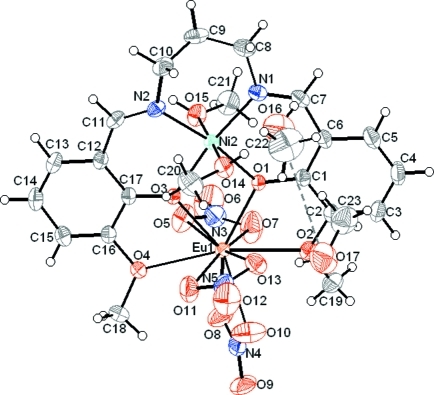
The molecular structure showing 40% probability displacement ellipsoids for non-H atoms. The dashed line indicates a hydrogen bond.

## supplementary crystallographic information

### Comment

As shown in Fig. 1, the hexadentate Schiff base ligand links Ni^II^ and Eu^III^ atoms into a dinuclear complex through two phenolate O atoms, which is the same as the bonding in the isostructural Ni^II^/Pr^III^ complex of the same ligand (Liu & Zhang, 2008) and a related Cu^II^/Sm^III^ complex (Wang *et al.*, 2008). The Eu^III^ ion is ten-coordinated by four O atoms from the ligand and six O atoms from three nitrate ions. The Ni^II^ ion is six-coordinated by two N atoms and two O atoms from the ligand and by two methanol molecules. They are two solvent methanol molecules for each complex. In the crystal structure, intermolecular O—H···O hydrogen bonds connect complexes and methanol solvent molecules to form chains along [010]. The chains can be viewed to lie in sheets in the (10\-2) planes.

### Experimental

The title complex was obtained by reaction of nickel(II) acetate tetrahydrate (0.0622 g, 0.25 mmol) with the Schiff base (0.0855 g, 0.25 mmol) in methanol (25 ml) at room temperature. Europium(III) nitrate hexahydrate (0.1117 g, 0.25 mmol) was added and the mixture was refluxed for 3 h. The reaction mixture was then cooled and filtered, and diethyl ether was allowed to diffuse slowly into the solution of the filtrate. Blue crystals were obtained after several days. Elemental analysis calculated; C, 31.93; H, 4.19; N, 8.09; found: C, 31.99; H, 4.20; N, 8.11.

### Refinement

H atoms bound to C atoms were placed in calculated positions and treated as riding on their parent atoms, with C—H = 0.93 Å (aromatic C), 0.97 Å (methylene C) or C—H = 0.96 Å (methyl C), and with *U*_iso_(H) = 1.2*U*_eq_(C) or 1.5*U*_eq_(C). H atoms bond to O atoms of uncoordinated methanol were placed in calculated positions and treated as riding on their parent atoms, with O—H = 0.82 Å, and with *U*_iso_(H) = 1.5*U*_eq_(O). H atoms bond to O atoms of coordinated methanol were initially located in a difference Fourier map and refined using restraints on the bond lengths (O—H = 0.82 (1) Å), after which they were constrained to ride on O with *U*_iso_(H) = 1.5*U*_eq_(O).

### Crystal data

**Table:**

[EuNi(C_19_H_20_N_2_O_4_)(NO_3_)_3_(CH_4_O)_2_]·2CH_4_OH	*F*(000) = 1744
*M**_r_* = 865.24	*D*_x_ = 1.791 Mg m^−^^3^
Monoclinic, *P*2_1_/*c*	Mo *K*α radiation, λ = 0.71073 Å
Hall symbol: -P 2ybc	Cell parameters from 25476 reflections
*a* = 13.062 (3) Å	θ = 3.0–27.5°
*b* = 11.105 (2) Å	µ = 2.61 mm^−^^1^
*c* = 22.122 (4) Å	*T* = 291 K
β = 90.81 (3)°	Block, brown
*V* = 3208.3 (11) Å^3^	0.24 × 0.23 × 0.21 mm
*Z* = 4	

### Data collection

**Table:**

Rigaku R-AXIS RAPID diffractometer	7257 independent reflections
Radiation source: fine-focus sealed tube	6231 reflections with *I* > 2σ(*I*)
graphite	*R*_int_ = 0.031
ω scan	θ_max_ = 27.5°, θ_min_ = 3.0°
Absorption correction: multi-scan (*ABSCOR*; Higashi, 1995)	*h* = −16→16
*T*_min_ = 0.572, *T*_max_ = 0.605	*k* = −14→13
28507 measured reflections	*l* = −28→28

### Refinement

**Table:**

Refinement on *F*^2^	Primary atom site location: structure-invariant direct methods
Least-squares matrix: full	Secondary atom site location: difference Fourier map
*R*[*F*^2^ > 2σ(*F*^2^)] = 0.028	Hydrogen site location: inferred from neighbouring sites
*wR*(*F*^2^) = 0.060	H-atom parameters constrained
*S* = 1.08	*w* = 1/[σ^2^(*F*_o_^2^) + (0.0114*P*)^2^ + 4.9558*P*] where *P* = (*F*_o_^2^ + 2*F*_c_^2^)/3
7257 reflections	(Δ/σ)_max_ = 0.027
430 parameters	Δρ_max_ = 0.65 e Å^−^^3^
6 restraints	Δρ_min_ = −0.46 e Å^−^^3^

### Special details

**Table:**

Geometry. All e.s.d.'s (except the e.s.d. in the dihedral angle between two l.s. planes) are estimated using the full covariance matrix. The cell e.s.d.'s are taken into account individually in the estimation of e.s.d.'s in distances, angles and torsion angles; correlations between e.s.d.'s in cell parameters are only used when they are defined by crystal symmetry. An approximate (isotropic) treatment of cell e.s.d.'s is used for estimating e.s.d.'s involving l.s. planes.
Refinement. Refinement of *F*^2^ against ALL reflections. The weighted *R*-factor *wR* and goodness of fit *S* are based on *F*^2^, conventional *R*-factors *R* are based on *F*, with *F* set to zero for negative *F*^2^. The threshold expression of *F*^2^ > σ(*F*^2^) is used only for calculating *R*-factors(gt) *etc*. and is not relevant to the choice of reflections for refinement. *R*-factors based on *F*^2^ are statistically about twice as large as those based on *F*, and *R*- factors based on ALL data will be even larger.

### Fractional atomic coordinates and isotropic or equivalent isotropic displacement parameters (Å^2^)

**Table:**

	*x*	*y*	*z*	*U*_iso_*/*U*_eq_	
C1	0.3764 (2)	0.4308 (3)	0.09086 (13)	0.0301 (6)	
C2	0.4448 (2)	0.3501 (3)	0.06332 (13)	0.0334 (6)	
C3	0.5468 (3)	0.3765 (3)	0.05608 (16)	0.0446 (8)	
H3	0.5899	0.3216	0.0374	0.054*	
C4	0.5848 (3)	0.4853 (4)	0.07678 (18)	0.0529 (9)	
H4	0.6539	0.5035	0.0728	0.064*	
C5	0.5204 (3)	0.5663 (3)	0.10317 (17)	0.0480 (9)	
H5	0.5465	0.6396	0.1167	0.058*	
C6	0.4157 (2)	0.5417 (3)	0.11034 (14)	0.0349 (7)	
C7	0.3548 (3)	0.6359 (3)	0.13797 (14)	0.0372 (7)	
H7	0.3882	0.7087	0.1447	0.045*	
C8	0.2244 (3)	0.7419 (3)	0.18478 (18)	0.0518 (9)	
H8A	0.2507	0.8117	0.1637	0.062*	
H8B	0.2523	0.7437	0.2257	0.062*	
C9	0.1101 (3)	0.7518 (3)	0.18760 (17)	0.0509 (9)	
H9A	0.0823	0.7489	0.1467	0.061*	
H9B	0.0928	0.8297	0.2045	0.061*	
C10	0.0591 (3)	0.6550 (3)	0.22458 (16)	0.0479 (9)	
H10A	0.0993	0.6406	0.2611	0.057*	
H10B	−0.0082	0.6825	0.2365	0.057*	
C11	−0.0400 (3)	0.4959 (3)	0.18735 (13)	0.0361 (7)	
H11	−0.0923	0.5380	0.2061	0.043*	
C12	−0.0689 (2)	0.3843 (3)	0.15762 (13)	0.0316 (6)	
C13	−0.1738 (3)	0.3570 (3)	0.15506 (15)	0.0416 (8)	
H13	−0.2203	0.4118	0.1707	0.050*	
C14	−0.2094 (3)	0.2525 (3)	0.13036 (16)	0.0464 (8)	
H14	−0.2793	0.2372	0.1283	0.056*	
C15	−0.1400 (3)	0.1689 (3)	0.10823 (15)	0.0407 (7)	
H15	−0.1634	0.0965	0.0920	0.049*	
C16	−0.0375 (2)	0.1932 (3)	0.11034 (13)	0.0311 (6)	
C17	0.0014 (2)	0.3024 (3)	0.13360 (12)	0.0287 (6)	
C18	0.0093 (3)	−0.0093 (3)	0.0860 (2)	0.0589 (11)	
H18A	−0.0231	−0.0331	0.1228	0.088*	
H18B	0.0695	−0.0572	0.0801	0.088*	
H18C	−0.0373	−0.0209	0.0526	0.088*	
C19	0.4543 (3)	0.1704 (3)	0.00302 (19)	0.0583 (11)	
H19A	0.4822	0.2207	−0.0279	0.087*	
H19B	0.4084	0.1127	−0.0150	0.087*	
H19C	0.5088	0.1289	0.0238	0.087*	
C20	0.1851 (4)	0.3577 (4)	0.27402 (18)	0.0688 (12)	
H20A	0.1583	0.4157	0.3018	0.103*	
H20B	0.2307	0.3041	0.2953	0.103*	
H20C	0.1298	0.3123	0.2563	0.103*	
C21	0.1495 (3)	0.5974 (4)	0.01424 (17)	0.0606 (11)	
H21A	0.1622	0.6816	0.0208	0.091*	
H21B	0.1068	0.5872	−0.0210	0.091*	
H21C	0.2134	0.5564	0.0085	0.091*	
C22	0.4141 (5)	0.4996 (6)	0.3499 (3)	0.117 (2)	
H22A	0.4814	0.5328	0.3558	0.176*	
H22B	0.4184	0.4134	0.3485	0.176*	
H22C	0.3713	0.5235	0.3827	0.176*	
C23	0.5326 (4)	0.3315 (5)	0.2241 (2)	0.0840 (15)	
H23A	0.4748	0.3845	0.2268	0.126*	
H23B	0.5508	0.3218	0.1825	0.126*	
H23C	0.5894	0.3651	0.2463	0.126*	
Eu1	0.216548 (11)	0.204199 (13)	0.078316 (7)	0.02910 (5)	
N1	0.2616 (2)	0.6317 (2)	0.15424 (11)	0.0350 (6)	
N2	0.0487 (2)	0.5428 (2)	0.19071 (11)	0.0340 (6)	
N3	0.1437 (3)	0.2941 (3)	−0.04323 (15)	0.0535 (8)	
N4	0.2728 (3)	−0.0388 (3)	0.03324 (16)	0.0546 (8)	
N5	0.2750 (3)	0.1093 (3)	0.19951 (14)	0.0504 (7)	
Ni2	0.17214 (3)	0.48224 (3)	0.146065 (16)	0.02754 (8)	
O1	0.27914 (15)	0.39775 (17)	0.09610 (9)	0.0299 (4)	
O2	0.39949 (17)	0.2432 (2)	0.04516 (10)	0.0395 (5)	
O3	0.10207 (15)	0.32092 (17)	0.13245 (9)	0.0298 (4)	
O4	0.03726 (17)	0.11435 (18)	0.08970 (10)	0.0383 (5)	
O5	0.0877 (2)	0.2728 (3)	0.00001 (12)	0.0673 (8)	
O6	0.1081 (3)	0.3307 (4)	−0.09113 (14)	0.0957 (12)	
O7	0.2364 (3)	0.2816 (3)	−0.03296 (15)	0.0853 (10)	
O8	0.2135 (2)	0.0327 (3)	0.00601 (15)	0.0739 (9)	
O9	0.2952 (3)	−0.1375 (3)	0.01348 (18)	0.0885 (11)	
O10	0.3103 (3)	−0.0001 (3)	0.08092 (15)	0.0849 (11)	
O11	0.1898 (2)	0.0829 (3)	0.17700 (13)	0.0644 (8)	
O12	0.3047 (3)	0.0714 (4)	0.24791 (15)	0.0928 (12)	
O13	0.3302 (2)	0.1800 (2)	0.16903 (11)	0.0492 (6)	
O14	0.23890 (18)	0.4177 (2)	0.22810 (10)	0.0439 (5)	
H24	0.2854	0.4608	0.2416	0.066*	
O15	0.10003 (18)	0.5488 (2)	0.06494 (10)	0.0449 (6)	
H25	0.0429	0.5769	0.0705	0.067*	
O16	0.3719 (3)	0.5426 (3)	0.29502 (15)	0.0917 (11)	
H16	0.3967	0.6087	0.2875	0.138*	
O17	0.5081 (3)	0.2221 (4)	0.2479 (2)	0.1067 (13)	
H17	0.4523	0.2005	0.2342	0.160*	

### Atomic displacement parameters (Å^2^)

**Table:**

	*U*^11^	*U*^22^	*U*^33^	*U*^12^	*U*^13^	*U*^23^
C1	0.0254 (16)	0.0353 (15)	0.0295 (14)	−0.0023 (12)	−0.0014 (11)	0.0045 (12)
C2	0.0265 (16)	0.0393 (16)	0.0345 (16)	−0.0015 (13)	0.0010 (12)	0.0048 (12)
C3	0.0278 (19)	0.055 (2)	0.051 (2)	0.0026 (15)	0.0048 (14)	0.0043 (16)
C4	0.0267 (19)	0.066 (2)	0.066 (2)	−0.0086 (17)	0.0055 (16)	0.0085 (19)
C5	0.037 (2)	0.049 (2)	0.057 (2)	−0.0148 (16)	−0.0030 (16)	0.0055 (16)
C6	0.0299 (17)	0.0372 (16)	0.0376 (16)	−0.0062 (13)	0.0001 (13)	0.0039 (13)
C7	0.041 (2)	0.0316 (15)	0.0389 (17)	−0.0089 (13)	−0.0052 (14)	0.0015 (12)
C8	0.058 (3)	0.0345 (17)	0.063 (2)	−0.0054 (16)	0.0022 (19)	−0.0141 (16)
C9	0.067 (3)	0.0318 (16)	0.054 (2)	0.0127 (17)	−0.0067 (19)	−0.0109 (15)
C10	0.048 (2)	0.0453 (19)	0.050 (2)	0.0059 (16)	0.0060 (16)	−0.0196 (16)
C11	0.0356 (19)	0.0400 (17)	0.0329 (16)	0.0119 (14)	0.0049 (13)	−0.0015 (13)
C12	0.0260 (16)	0.0394 (16)	0.0294 (14)	0.0023 (12)	0.0041 (11)	0.0036 (12)
C13	0.0277 (18)	0.054 (2)	0.0428 (18)	0.0075 (15)	0.0044 (14)	0.0021 (15)
C14	0.0249 (18)	0.062 (2)	0.052 (2)	−0.0051 (15)	−0.0003 (14)	0.0022 (17)
C15	0.0329 (19)	0.0451 (18)	0.0441 (18)	−0.0089 (14)	−0.0036 (14)	−0.0009 (14)
C16	0.0266 (16)	0.0355 (15)	0.0313 (14)	−0.0009 (12)	0.0010 (11)	0.0029 (12)
C17	0.0247 (15)	0.0347 (14)	0.0265 (13)	−0.0005 (12)	−0.0003 (10)	0.0053 (12)
C18	0.052 (3)	0.0288 (17)	0.096 (3)	−0.0065 (16)	0.011 (2)	−0.0027 (18)
C19	0.056 (3)	0.050 (2)	0.069 (3)	0.0074 (18)	0.026 (2)	−0.0120 (18)
C20	0.083 (3)	0.072 (3)	0.051 (2)	−0.015 (2)	0.002 (2)	0.017 (2)
C21	0.065 (3)	0.072 (3)	0.046 (2)	0.005 (2)	0.0038 (19)	0.0201 (19)
C22	0.125 (5)	0.137 (5)	0.088 (4)	0.018 (4)	−0.040 (4)	−0.011 (4)
C23	0.076 (4)	0.092 (4)	0.084 (4)	0.014 (3)	−0.009 (3)	0.016 (3)
Eu1	0.02479 (8)	0.02687 (7)	0.03562 (8)	0.00140 (6)	−0.00035 (5)	−0.00468 (6)
N1	0.0395 (16)	0.0293 (13)	0.0360 (14)	−0.0023 (11)	−0.0022 (11)	−0.0024 (10)
N2	0.0356 (16)	0.0347 (13)	0.0317 (13)	0.0067 (11)	0.0016 (11)	−0.0073 (10)
N3	0.049 (2)	0.0567 (19)	0.0542 (19)	0.0025 (16)	−0.0132 (15)	0.0090 (15)
N4	0.042 (2)	0.0444 (17)	0.078 (2)	−0.0082 (14)	0.0140 (16)	−0.0221 (16)
N5	0.045 (2)	0.0552 (18)	0.0512 (18)	0.0093 (15)	−0.0013 (14)	0.0119 (15)
Ni2	0.0274 (2)	0.02572 (17)	0.02952 (18)	0.00102 (14)	0.00060 (14)	−0.00302 (14)
O1	0.0226 (11)	0.0294 (10)	0.0377 (11)	−0.0021 (8)	0.0029 (8)	−0.0042 (8)
O2	0.0291 (12)	0.0406 (12)	0.0491 (13)	0.0022 (9)	0.0089 (10)	−0.0092 (10)
O3	0.0229 (11)	0.0289 (10)	0.0377 (11)	−0.0011 (8)	0.0021 (8)	−0.0047 (8)
O4	0.0310 (13)	0.0302 (11)	0.0538 (14)	−0.0045 (9)	0.0017 (10)	−0.0053 (9)
O5	0.0567 (19)	0.096 (2)	0.0494 (16)	0.0160 (16)	0.0028 (13)	0.0104 (15)
O6	0.091 (3)	0.138 (3)	0.0573 (19)	0.017 (2)	−0.0185 (18)	0.042 (2)
O7	0.053 (2)	0.120 (3)	0.082 (2)	0.0030 (19)	−0.0047 (16)	0.034 (2)
O8	0.065 (2)	0.0622 (18)	0.094 (2)	0.0188 (16)	−0.0173 (17)	−0.0380 (17)
O9	0.068 (2)	0.0525 (17)	0.145 (3)	0.0137 (15)	0.000 (2)	−0.0510 (19)
O10	0.128 (3)	0.0564 (18)	0.070 (2)	0.0237 (19)	−0.017 (2)	−0.0108 (16)
O11	0.0397 (17)	0.0777 (19)	0.0756 (19)	−0.0027 (14)	−0.0029 (14)	0.0263 (16)
O12	0.084 (3)	0.126 (3)	0.068 (2)	0.009 (2)	−0.0138 (18)	0.051 (2)
O13	0.0516 (16)	0.0457 (14)	0.0501 (14)	−0.0071 (11)	−0.0095 (12)	0.0075 (11)
O14	0.0435 (15)	0.0486 (13)	0.0394 (12)	−0.0047 (11)	−0.0051 (10)	0.0071 (10)
O15	0.0386 (14)	0.0582 (15)	0.0378 (12)	0.0057 (11)	−0.0013 (10)	0.0110 (11)
O16	0.100 (3)	0.090 (2)	0.084 (2)	−0.005 (2)	−0.045 (2)	−0.0049 (19)
O17	0.077 (3)	0.108 (3)	0.134 (3)	−0.012 (2)	−0.037 (2)	0.010 (3)

### Geometric parameters (Å, °)

**Table:**

C1—O1	1.329 (3)	C20—O14	1.410 (4)
C1—C6	1.401 (4)	C20—H20A	0.960
C1—C2	1.410 (4)	C20—H20B	0.960
C2—C3	1.376 (4)	C20—H20C	0.960
C2—O2	1.384 (4)	C21—O15	1.410 (4)
C3—C4	1.382 (5)	C21—H21A	0.960
C3—H3	0.930	C21—H21B	0.960
C4—C5	1.368 (5)	C21—H21C	0.960
C4—H4	0.930	C22—O16	1.409 (6)
C5—C6	1.406 (5)	C22—H22A	0.960
C5—H5	0.930	C22—H22B	0.960
C6—C7	1.453 (4)	C22—H22C	0.960
C7—N1	1.275 (4)	C23—O17	1.364 (6)
C7—H7	0.930	C23—H23A	0.960
C8—N1	1.483 (4)	C23—H23B	0.960
C8—C9	1.500 (5)	C23—H23C	0.960
C8—H8A	0.970	Eu1—O3	2.3239 (19)
C8—H8B	0.970	Eu1—O1	2.3311 (19)
C9—C10	1.511 (5)	Eu1—O8	2.487 (3)
C9—H9A	0.970	Eu1—O13	2.494 (3)
C9—H9B	0.970	Eu1—O5	2.516 (3)
C10—N2	1.460 (4)	Eu1—O2	2.546 (2)
C10—H10A	0.970	Eu1—O4	2.561 (2)
C10—H10B	0.970	Eu1—O10	2.579 (3)
C11—N2	1.271 (4)	Eu1—O11	2.593 (3)
C11—C12	1.451 (4)	Eu1—O7	2.624 (3)
C11—H11	0.930	Eu1—Ni2	3.4844 (7)
C12—C17	1.401 (4)	N1—Ni2	2.036 (3)
C12—C13	1.403 (4)	N2—Ni2	2.017 (3)
C13—C14	1.362 (5)	N3—O6	1.221 (4)
C13—H13	0.930	N3—O5	1.235 (4)
C14—C15	1.391 (5)	N3—O7	1.237 (4)
C14—H14	0.930	N4—O9	1.216 (4)
C15—C16	1.366 (4)	N4—O10	1.234 (4)
C15—H15	0.930	N4—O8	1.257 (4)
C16—O4	1.393 (4)	N5—O12	1.210 (4)
C16—C17	1.409 (4)	N5—O11	1.247 (4)
C17—O3	1.332 (3)	N5—O13	1.267 (4)
C18—O4	1.422 (4)	Ni2—O1	2.025 (2)
C18—H18A	0.960	Ni2—O3	2.0322 (19)
C18—H18B	0.960	Ni2—O14	2.127 (2)
C18—H18C	0.960	Ni2—O15	2.146 (2)
C19—O2	1.433 (4)	O14—H24	0.826
C19—H19A	0.960	O15—H25	0.820
C19—H19B	0.960	O16—H16	0.820
C19—H19C	0.960	O17—H17	0.820
			
O1—C1—C6	124.2 (3)	O3—Eu1—O13	91.46 (8)
O1—C1—C2	118.4 (3)	O1—Eu1—O13	76.11 (7)
C6—C1—C2	117.4 (3)	O8—Eu1—O13	116.05 (9)
C3—C2—O2	124.1 (3)	O3—Eu1—O5	75.94 (8)
C3—C2—C1	122.4 (3)	O1—Eu1—O5	93.82 (9)
O2—C2—C1	113.5 (3)	O8—Eu1—O5	77.54 (10)
C2—C3—C4	119.4 (3)	O13—Eu1—O5	166.27 (9)
C2—C3—H3	120.3	O3—Eu1—O2	131.83 (7)
C4—C3—H3	120.3	O1—Eu1—O2	64.13 (7)
C5—C4—C3	119.8 (3)	O8—Eu1—O2	87.23 (9)
C5—C4—H4	120.1	O13—Eu1—O2	72.52 (8)
C3—C4—H4	120.1	O5—Eu1—O2	111.80 (9)
C4—C5—C6	121.7 (3)	O3—Eu1—O4	64.58 (7)
C4—C5—H5	119.2	O1—Eu1—O4	131.42 (7)
C6—C5—H5	119.2	O8—Eu1—O4	76.01 (9)
C1—C6—C5	119.3 (3)	O13—Eu1—O4	114.44 (8)
C1—C6—C7	124.2 (3)	O5—Eu1—O4	65.30 (9)
C5—C6—C7	116.5 (3)	O2—Eu1—O4	163.24 (7)
N1—C7—C6	128.5 (3)	O3—Eu1—O10	142.01 (9)
N1—C7—H7	115.7	O1—Eu1—O10	129.96 (10)
C6—C7—H7	115.7	O8—Eu1—O10	49.00 (10)
N1—C8—C9	114.3 (3)	O13—Eu1—O10	67.06 (9)
N1—C8—H8A	108.7	O5—Eu1—O10	126.47 (10)
C9—C8—H8A	108.7	O2—Eu1—O10	73.07 (10)
N1—C8—H8B	108.7	O4—Eu1—O10	95.14 (10)
C9—C8—H8B	108.7	O3—Eu1—O11	76.15 (8)
H8A—C8—H8B	107.6	O1—Eu1—O11	112.83 (9)
C8—C9—C10	114.7 (3)	O8—Eu1—O11	98.19 (11)
C8—C9—H9A	108.6	O13—Eu1—O11	49.63 (9)
C10—C9—H9A	108.6	O5—Eu1—O11	129.81 (9)
C8—C9—H9B	108.6	O2—Eu1—O11	117.96 (8)
C10—C9—H9B	108.6	O4—Eu1—O11	65.21 (8)
H9A—C9—H9B	107.6	O10—Eu1—O11	66.02 (10)
N2—C10—C9	111.6 (3)	O3—Eu1—O7	111.94 (10)
N2—C10—H10A	109.3	O1—Eu1—O7	79.45 (10)
C9—C10—H10A	109.3	O8—Eu1—O7	69.43 (12)
N2—C10—H10B	109.3	O13—Eu1—O7	136.42 (10)
C9—C10—H10B	109.3	O5—Eu1—O7	47.85 (10)
H10A—C10—H10B	108.0	O2—Eu1—O7	64.42 (9)
N2—C11—C12	127.4 (3)	O4—Eu1—O7	108.83 (9)
N2—C11—H11	116.3	O10—Eu1—O7	104.84 (11)
C12—C11—H11	116.3	O11—Eu1—O7	167.53 (11)
C17—C12—C13	119.3 (3)	C7—N1—C8	114.7 (3)
C17—C12—C11	124.0 (3)	C7—N1—Ni2	123.6 (2)
C13—C12—C11	116.8 (3)	C8—N1—Ni2	121.5 (2)
C14—C13—C12	121.9 (3)	C11—N2—C10	117.2 (3)
C14—C13—H13	119.1	C11—N2—Ni2	124.7 (2)
C12—C13—H13	119.1	C10—N2—Ni2	117.8 (2)
C13—C14—C15	119.3 (3)	O6—N3—O5	120.9 (4)
C13—C14—H14	120.3	O6—N3—O7	123.8 (4)
C15—C14—H14	120.3	O5—N3—O7	115.1 (3)
C16—C15—C14	119.9 (3)	O9—N4—O10	121.8 (4)
C16—C15—H15	120.0	O9—N4—O8	123.1 (4)
C14—C15—H15	120.0	O10—N4—O8	115.1 (3)
C15—C16—O4	123.8 (3)	O12—N5—O11	123.0 (3)
C15—C16—C17	122.0 (3)	O12—N5—O13	120.6 (4)
O4—C16—C17	114.2 (2)	O11—N5—O13	116.4 (3)
O3—C17—C12	124.0 (3)	N2—Ni2—O1	169.77 (9)
O3—C17—C16	118.5 (3)	N2—Ni2—O3	90.28 (9)
C12—C17—C16	117.5 (3)	O1—Ni2—O3	79.83 (8)
O4—C18—H18A	109.5	N2—Ni2—N1	98.42 (11)
O4—C18—H18B	109.5	O1—Ni2—N1	91.51 (9)
H18A—C18—H18B	109.5	O3—Ni2—N1	171.22 (9)
O4—C18—H18C	109.5	N2—Ni2—O14	90.88 (10)
H18A—C18—H18C	109.5	O1—Ni2—O14	91.82 (9)
H18B—C18—H18C	109.5	O3—Ni2—O14	90.43 (9)
O2—C19—H19A	109.5	N1—Ni2—O14	88.35 (10)
O2—C19—H19B	109.5	N2—Ni2—O15	87.17 (10)
H19A—C19—H19B	109.5	O1—Ni2—O15	90.06 (9)
O2—C19—H19C	109.5	O3—Ni2—O15	89.32 (9)
H19A—C19—H19C	109.5	N1—Ni2—O15	92.19 (10)
H19B—C19—H19C	109.5	O14—Ni2—O15	178.03 (9)
O14—C20—H20A	109.5	C1—O1—Ni2	126.01 (18)
O14—C20—H20B	109.5	C1—O1—Eu1	124.97 (17)
H20A—C20—H20B	109.5	Ni2—O1—Eu1	106.04 (8)
O14—C20—H20C	109.5	C2—O2—C19	117.2 (3)
H20A—C20—H20C	109.5	C2—O2—Eu1	117.49 (17)
H20B—C20—H20C	109.5	C19—O2—Eu1	124.8 (2)
O15—C21—H21A	109.5	C17—O3—Ni2	125.17 (17)
O15—C21—H21B	109.5	C17—O3—Eu1	124.52 (17)
H21A—C21—H21B	109.5	Ni2—O3—Eu1	106.04 (8)
O15—C21—H21C	109.5	C16—O4—C18	116.4 (3)
H21A—C21—H21C	109.5	C16—O4—Eu1	115.67 (16)
H21B—C21—H21C	109.5	C18—O4—Eu1	127.2 (2)
O16—C22—H22A	109.5	N3—O5—Eu1	101.2 (2)
O16—C22—H22B	109.5	N3—O7—Eu1	95.7 (2)
H22A—C22—H22B	109.5	N4—O8—Eu1	99.9 (2)
O16—C22—H22C	109.5	N4—O10—Eu1	96.0 (2)
H22A—C22—H22C	109.5	N5—O11—Eu1	94.8 (2)
H22B—C22—H22C	109.5	N5—O13—Eu1	99.1 (2)
O17—C23—H23A	109.5	C20—O14—Ni2	124.9 (2)
O17—C23—H23B	109.5	C20—O14—H24	112.6
H23A—C23—H23B	109.5	Ni2—O14—H24	113.8
O17—C23—H23C	109.5	C21—O15—Ni2	126.6 (2)
H23A—C23—H23C	109.5	C21—O15—H25	113.7
H23B—C23—H23C	109.5	Ni2—O15—H25	113.4
O3—Eu1—O1	68.00 (7)	C22—O16—H16	109.1
O3—Eu1—O8	138.92 (9)	C23—O17—H17	109.3
O1—Eu1—O8	144.80 (10)		

### Hydrogen-bond geometry (Å, °)

**Table:**

*D*—H···*A*	*D*—H	H···*A*	*D*···*A*	*D*—H···*A*
O14—H24···O16	0.83	1.86	2.657 (4)	162
O15—H25···O6^i^	0.82	2.28	3.092 (4)	174
O16—H16···O17^ii^	0.82	1.94	2.715 (6)	157
O17—H17···O13	0.82	2.15	2.923 (5)	158

Symmetry codes: (i) −*x*, −*y*+1, −*z*; (ii) −*x*+1, *y*+1/2, −*z*+1/2.
